# From Searching to Coping, How Chinese Patients With Breast Cancer Navigate Web-Based Health Information: Semistructured Interview Study

**DOI:** 10.2196/80363

**Published:** 2026-03-26

**Authors:** Jiahui Liu, Xingfeng Li, Xuying Li, Jiejun Chen, Dan Zhang, Cuiling Qiu

**Affiliations:** 1 Department of Breast Medicine, Hunan Cancer Hospital, 582 Xianjiahu Road, Yuelu District, Changsha, Hunan, 410013, China, 86 15116394002; 2 Department of Nursing, Hunan Cancer Hospital, 582 Xianjiahu Road, Yuelu District, Changsha, Hunan, 410013, China, 86 15116394002

**Keywords:** breast cancer, information seeking, decision-making, coping strategy, qualitative research

## Abstract

**Background:**

With the development of digital health platforms, patients with breast cancer are increasingly relying on web-based resources to search for disease-related information. Proper usage of web-based health information by patients with breast cancer is crucial for understanding disease information and participating in treatment decisions. However, in the face of the large amount and complexity of information, it is still unclear how patients can make psychological adjustments and behavioral responses. Problems such as variable information quality and conflicting information are also affecting the cognitive and treatment decision-making process of patients with breast cancer.

**Objective:**

This study aims to explore the real experiences of Chinese patients with breast cancer in their search for web-based health information from a phenomenological perspective, providing insights for optimizing future web-based health information support for patients.

**Methods:**

This qualitative study used semistructured, in-depth face-to-face interviews to collect data. Through purposive and convenience sampling, 18 female patients with breast cancer were recruited from a tertiary cancer hospital in China. The data saturation principle was observed to determine the endpoint of data collection. The collected data were analyzed using thematic analysis.

**Results:**

From 18 original interview documents, three themes and 11 subthemes were categorized as follows: (1) driving force of information search (emotion-based information search, problem-solving–oriented information search), (2) cognitive judgments amidst the information fog (interweaving of multichannel information, judgment of information authenticity, information applicability assessment, cognitive confusion in the context of information conflict, and construction of information meaning), and (3) adaptation under the pressure of web-based information (transform information into action, emotional regulatory coping, build a support network, and acceptance and adjustment of expectations).

**Conclusions:**

This study reveals that the experiences of patients with breast cancer within web-based health information environments resemble an information navigation journey. Patients continuously search, evaluate, and adjust within the sea of information to maintain cognitive clarity and emotional equilibrium. The findings offer valuable insights for clinical health care providers, health information platform developers, and policymakers. They can help optimize digital health services and design personalized information support that better meets patients’ needs.

## Introduction

According to the latest global cancer data in 2020, breast cancer has surpassed lung cancer to become the world’s leading cancer, being the main cause of cancer incidence, disability, and mortality among women worldwide, imposing a huge burden on global health services [1]. With the advancement of medical technology and the promotion of early screening, more and more patients with breast cancer can receive early diagnosis and treatment, and their survival prognosis has significantly improved compared with before [2,3]. However, due to the complex pathological characteristics of breast cancer, diverse treatment options, and long-term rehabilitation needs, patients’ demand for health information is extremely urgent. A survey study found that among the patients with breast cancer interviewed, as many as 85% expressed a desire to obtain more information about their disease [4]. Sheehy et al [5] conducted a prospective longitudinal investigation and found that the information needs of patients with breast cancer remained high throughout the entire follow-up period of 1 year, 3 years, and 5 years after diagnosis. The research conducted by Ludwigson et al [6] revealed that patients with breast cancer were dissatisfied with the health information they received regarding disease prognosis, symptom management, and surgical reconstruction. It can be seen that patients with breast cancer have long-term demands for health information, but these demands have not been met.

Therefore, with the rapid development of the internet and the increasing inclusiveness, accessibility, and comprehensibility of digital media, an increasing number of patients are beginning to actively seek health information through web-based channels to assist in their treatment decisions [7]. It has been reported that the internet has become the preferred source of information for patients with cancer, surpassing health care professionals [8]. According to relevant research reports, breast cancer is one of the most frequently searched cancer topics on the internet [9]. Online health information search (OHIS) plays a significant role in the health management of patients with chronic diseases, enabling them to understand their conditions and facilitating their participation in the decision-making process regarding treatment [10,11].

However, the current digital health information environment also presents numerous challenges. Given the large amount of inaccurate web-based information, people can easily become misinformed [12]. A study has shown that misleading web-based health information is associated with poor patient outcomes [13]. Moreover, there is an abundance of web-based health information, but the quality of this information is difficult to guarantee [14]. In the face of the challenges brought by web-based information, patients with breast cancer often need to make psychological trade-offs, forming a complex coping mechanism. The “Stress and Coping Theory” proposed by Lazarus and Folkman [15] provides a theoretical framework for understanding this phenomenon. According to this theory, when individuals encounter potentially threatening events, they undergo primary appraisal and secondary appraisal and, based on the situation, choose appropriate coping strategies [16]. This theory proposes two primary coping strategies: (1) problem-focused coping, aimed at resolving the problem or situation; and (2) emotion-focused/avoidance coping, aimed at regulating stress-related emotions [17]. Problem-focused coping involves acquiring relevant information, while emotion-focused coping entails avoiding thoughts about the threat or reevaluating the threat without altering the stressful reality. These 2 coping strategies may either promote or mutually hinder each other in stressful situations [18]. This theory has been widely applied to analyze the psychological adaptation mechanisms of patients in disease management and health behavior decision-making [19,20].

Although some studies have preliminarily explored the OHIS behaviors of patients with breast cancer, limited research has explored their subjective experiences, cognitive processes, and coping strategies—particularly within the Chinese cultural context [8,21]. Most of the existing literature focuses on the objective characteristics of information behavior. The perspective of phenomenological research emphasizes the genuine understanding of individual experiences [22]. Therefore, this study aims to explore the experiences of patients with breast cancer in web-based health information contexts from a phenomenological perspective. To facilitate a deeper understanding of the psychological mechanisms reflected in participants’ experiences, the Stress and Coping Theory was drawn upon as an interpretive reference. By filling this underexplored research gap, this study has deepened our understanding of how breast cancer patients search for, evaluate, and apply health information in a complex information environment. The research results provide practical guidance for designing personalized and operational web-based health interventions and information support strategies in clinical practice and may also offer insights for the development of digital health services for other chronic diseases or vulnerable groups.

## Methods

### Study Design

Guided by a phenomenological perspective and using qualitative research methods, this study aims to explore in depth the experiences and feelings of Chinese patients with breast cancer as they search for web-based health information during their disease journey [22]. Data were collected through semistructured face-to-face in-depth interviews. This study drew upon Lazarus and Folkman’s [15] stress-coping theory in designing the interview guide. Following thematic analysis, researchers used this theoretical framework to interpret patients’ cognitive evaluations and coping strategies within their web-based information-seeking behaviors, thereby enhancing the theoretical significance of the findings. Originally proposed in 1984, this classic psychological theory explains how individuals assess and respond to stressful events through a dynamic regulatory process involving person–environment interactions. The study reporting followed the COREQ (Consolidated Criteria for Reporting Qualitative Research Checklist; Checklist 1) [23].

### Setting and Participants

From September 2024 to May 2025, participants were recruited through a combination of convenience and purposive sampling in 3 breast cancer inpatient wards at Hunan Cancer Hospital in China. Hunan Province is a typical province in central China, comprising 14 prefecture-level cities. Its diversity in language and culture, urban-rural structure, health care resources, economic development, and residents’ digital literacy provides a suitable setting for examining the contextual variations in information-seeking behaviors [24].

Two female researchers (LJH and ZD) were Advanced Practice Nurses in breast oncology with training in breast disease management and qualitative interviewing. The researcher has experience practicing in participants’ workplaces and is responsible for participant screening and interview scheduling. First, based on convenience sampling principles, the researcher reviewed daily inpatient records and made preliminary assessments of eligibility based on medical documentation. Subsequently, clinicians and charge nurses assisted in notifying eligible patients. Researchers then conducted face-to-face interactions with patients, explaining the study objectives, procedures, timeline, and privacy safeguards to invite participation. To enrich the data dimensions, the research team purposefully included patients of varying ages, educational levels, disease stages, duration since diagnosis, and urban/rural residence after initial recruitment, continuing until data saturation was achieved.

### Participant Recruitment and Enrollment

Participants were female patients diagnosed with breast cancer who had experience in searching for information on the internet. Inclusion criteria included the following: (1) aged 18 years and older; (2) diagnosed with breast cancer; (3) able to express themselves clearly, willing to participate in the study, and sign an informed consent form; and (4) previously actively searched for health information on the internet or mobile platforms during the disease. Exclusion criteria included the following: (1) comorbid serious mental illness affecting the interview communication; (2) those who refused to record or were unable to complete the interview.

### Data Collection

Data were collected through semistructured, in-depth interviews. Based on the study aims, a review of relevant literature, and the theoretical framework of stress and coping, the interview guide was developed collaboratively by the research team members (LJH and QCL) with guidance from a qualitative research expert (LXY). To enhance clarity, exploratory questions were incorporated into the primary interview questions to better capture the depth and complexity of participants’ experiences, as shown in Table 1. Two participants who met the inclusion criteria were invited for a pilot interview. Based on the feedback from the pilot participants, the wording and sequence of the interview questions were adjusted, and the exploratory questions were also optimized.

**Table 1.** Interview guide.

Main question	Exploratory questions
a. Please tell us about your motivation and initial experience of online health information searching.	Under what circumstances did you begin using the internet to search for health information related to breast cancer? What kind of information were you initially looking for? What is the primary motivation for searching for information online?
b. Please tell us about the process of your information search.	Which platforms or websites do you typically use to access online health information? What challenges have you encountered during the information search process? How do you screen and evaluate online health information? Do you have any criteria or methods you rely on?
c. Please tell us about the impact of online health information on your emotions.	How do you feel when searching for and reading online health information? How has online health impacted you? Could you describe your emotional experience in more detail?
d. Please tell us about the impact of online health information on your treatment decision-making.	l How does online health information influence your treatment decisions? How do you think searching for health information online affects your communication with your doctor? Can you discuss how you question or offer suggestions to your doctor based on online health information?
e. Please tell us about your overall evaluation of and expectations for online health information.	What role do you think online health information played in your journey to overcome breast cancer? If given the opportunity, how would you improve your information-seeking process? What kind of assistance or support would you like to receive?

The interview began after obtaining the participants’ verbal and written informed consent. The entire process was audio-recorded after obtaining their consent, and at the same time, the researchers took field notes to capture nonverbal information and key immediate thoughts. During the interview, the researcher flexibly adjusted the order and depth of questions according to the participants’ expressions and responses and encouraged patients to describe their information-seeking experience in-depth and truthfully. The average duration of the interviews is approximately 45 minutes (range 20‐60 min). All interviews were conducted in quiet hospital spaces designated for patient–clinician communication to ensure privacy and comfort. To minimize interviewer bias and ensure the authenticity of participants’ expressions, the researcher adopted a neutral and nonevaluative stance during the interviews, avoided leading or suggestive questions, and respected each participant’s subjective experiences. When no new topics are generated, data collection is halted [25]. When no new significant themes emerge after analyzing 2 consecutive rounds of interviews, data saturation is achieved, and sampling ceases.

### Data Analysis

The interview recordings were transcribed verbatim into Chinese text by the researcher (LJH) within 24 hours of completion and verified for accuracy by the researcher (ZD). Transcribed texts are managed using Excel, with each record containing participant ID, raw text, open coding, thematic categorization, and researcher notes. Excel files are uniformly stored in a controlled environment and encrypted to ensure participant information security and privacy.

Using thematic analysis as the methodological basis for theme summarization [26]. Two researchers (LJH and ZD) independently conducted the initial coding and then discussed and reached a consensus. In case of disagreement, a third-party expert (LXF) was invited to participate in the discussion to ensure the reliability and authenticity of the analysis. After coding completion, the researcher (LJH) categorized and merged relevant codes to form preliminary themes. These themes underwent discussion and review by all researchers to ensure alignment with the original data. Subsequently, themes were defined and named, with descriptive explanations drafted for each. Finally, themes were linked to corresponding original data citations. The researchers provided the preliminary analysis results to some participants for feedback and solicited their opinions to ensure the authenticity and representativeness of the analysis. To ensure the accuracy of language conversion, all citations and theme descriptions intended for publication were translated from the original Chinese into English by professional bilingual personnel with medical backgrounds. Subsequently, all members of the research team conducted back-translation and concept [27].

### Reflexivity

Throughout the research process, the team maintained a reflective stance to enhance transparency, credibility, and rigor. Given that the interviewers had professional backgrounds in the breast department, face-to-face semistructured interviews fostered trust, enabling participants to be more willing to share their personal experiences. However, this also risked leading researchers to overinterpret participants’ perspectives during interviews, potentially overlooking subtle variations in their experiences. Therefore, researchers maintained reflective journals before and after each interview, documenting their preconceptions, emotional responses, interview interactions, and any subjective impressions that might influence interpretation. These records were repeatedly reviewed during subsequent analysis. During data analysis, researchers repeatedly returned to original recordings to ensure initial coding stemmed from participants’ authentic expressions. Researchers took particular care to separate clinical experience from the analytical process, avoiding the substitution of professional judgments for meanings presented in participants’ narratives. Throughout theme development, the research team continuously challenged, compared, and refined interpretations through ongoing peer discussions and reflective dialogues. This multiperspective examination of coding consistency and thematic robustness enhanced the credibility and confirmation of research findings. Additionally, the team deliberately revisited earlier hypotheses and analytical pathways during the later stages of analysis [28].

### Ethical Considerations

The study was approved by the Hunan Cancer Hospital Medical Ethics Review Committee (number SBQLL-2024‐180). All participants signed written informed consent forms prior to the commencement of face-to-face interviews, ensuring their participation was voluntary, information was anonymized, and data were used solely for this study. Furthermore, no financial compensation was provided to any participants. All raw interview data and participant information underwent anonymization and were stored on an encrypted cloud storage drive to ensure confidentiality.

## Results

### Demographic Characteristics

A total of 18 female patients with breast cancer participated, originating from Wuhan, Hubei Province (n=1), Jiamusi City, Heilongjiang Province (n=1), and 9 prefecture-level cities in Hunan Province (Changsha, Yiyang, Changde, Hengyang, Loudi, Zhuzhou, Shaoyang, Yongzhou, and Yueyang) (n=16). All the participants were of Han ethnicity. All patients with breast cancer are currently undergoing treatment in the hospital. Except for one participant who had bilateral breast cancer, all the other participants had unilateral breast cancer. The time of cancer diagnosis for the participants ranged from 1 month to 20 years. The most commonly used digital search platform by the participants was Baidu. The general information of the participants is shown in Table 2.

**Table 2.** Characteristics of the participants.

Variables	Participants (N=18)
Age (years), mean (SD)	42.29 (10.60)
Education background, n (%)	
Junior high school and below	5（27.8）
Senior high school	3（16.7）
Bachelor degree	8（44.4）
Graduate degree	2（11.1）
Employment, n (%)	
Employed	6（33.3）
Unemployed	10（55.6）
Retired	2（11.1）
Marital status, n (%)	
Unmarried	1（5.56）
Married	15（83.3）
Divorced	2（11.1）
Medical insurance payment methods, n (%)	
Employee Medical Insurance	12（66.7）
Resident Medical Insurance	3（16.7）
New Rural Cooperative Medical Scheme	3（16.7）
Year of breast cancer diagnosis, n (%)	
＜1	9（50.0）
1-4	4（22.2）
5-9	2（11.1）
＞10	3（16.7）
Clinical stages, n (%)	
Stage Ⅰ-II	9（50.0）
Stage Ⅲ-IV	9（50.0）
Relapse and metastasis, n (%)	
Yes	9（50.0）
No	9（50.0）
Digital seeking platform, n (%)	
Baidu^a^	15（83.3）
XiaoHongShu^b^	5（27.8）
TikTok^c^	7（38.9）
Deepseek^d^	7（38.9）
WeChat^e^	4（22.2）
TengXunYuanBao^d^	1（5.56）

a Baidu: Comprehensive search engine.

b Xiaohongshu: Social sharing platform primarily featuring user-generated content.

c TikTok: Short-video-centric information dissemination platform.

d DeepSeek and TengXunYuanBao: Artificial intelligence-based intelligent service platform.

e WeChat: Comprehensive social platform.

**Figure 1.**
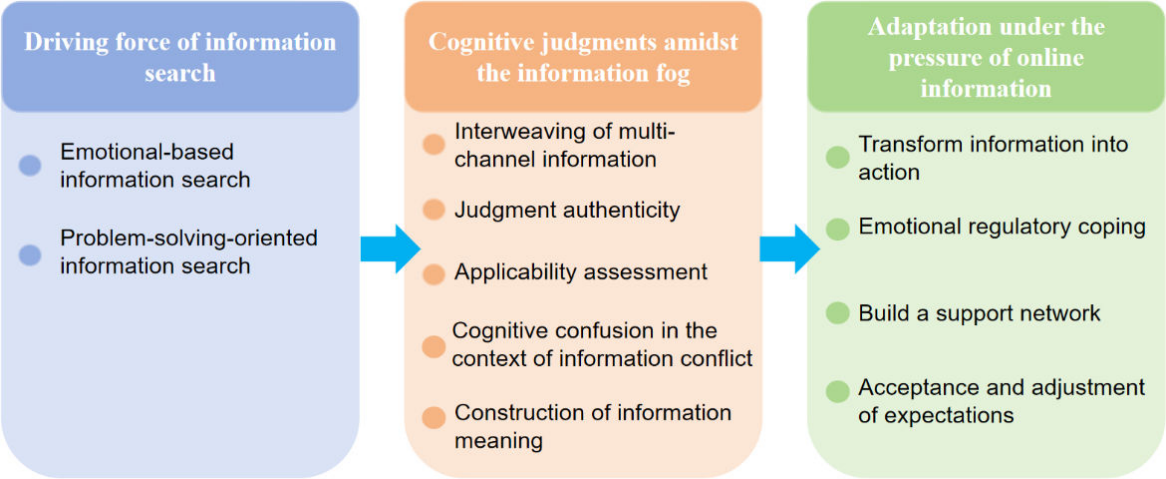
Coding framework of the web-based health information navigation process among patients with breast cancer.

This study identified three core themes and 11 subthemes of OHIS in patients with breast cancer through thematic analysis: (1) the driving forces of information search were emotion-driven and problem-oriented; (2) cognitive judgments in the information fog included the interweaving of multiple channels of information, assessment of information authenticity and applicability, cognitive confusion under information conflicts, and the construction of information meaning; (3) adaptive strategies under the pressure of network information encompassed information conversion into action, emotion regulation, support network construction, and expectation acceptance and adjustment. Overall, the OHIS behaviors of patients with breast cancer present a dynamic process driven by motivation, cognitive processing, and adaptive strategies. The overall coding framework, including the hierarchical relationship between themes and subthemes, is shown in Figure 1.

### Driving Force of Information Search

#### Overview

This theme reflected the multiple drivers behind information-seeking behaviors of patients with breast cancer, stemming from the combined influence of emotional needs and practical concerns. On one hand, information retrieval served to alleviate uncertainty and provide emotional reassurance; on the other, patients also used web-based resources to understand their disease, support treatment decisions, and address daily management needs.

#### Emotion-Based Information Search

The information-seeking behavior of participants was an emotionally driven experience process. It began with the anxiety, fear, and uncertainty triggered by the diagnosis, and the participants attempted to obtain psychological comfort and a sense of control through searching.

*I just wanna see if it’s really breast cancer. It’s just for some peace of mind. If it’s not, I'll definitely feel better, right? I'm also really scared about myself…*

[P6]

*That kind of bad news really makes me anxious… Especially when I see stuff about having to take medicine long - term and go through all these treatments. It makes me super anxious, like the treatments just never end!*

[P5]

*I'm worried if there’s still hope for a cure… 'Cause I've got old folks to take care of and kids to raise. Wanna check if there are any such cases…*

[P1]

However, web-based health information not only serves a knowledge function; its content itself could directly influence patients’ emotional states, becoming a significant trigger that amplifies positive or negative emotions. When search results were interpreted as “positive,” patients often experienced a brief sense of hope and emotional uplift; conversely, negative or uncertain information could significantly exacerbate anxiety and emotional distress.

*If the search results are good, I'll feel a bit happier. If they're bad, I'll definitely worry more.*

[P6]

*When you see something good, you'll feel great. But when you see something bad, it'll affect you. Your mood can easily be influenced by the internet. Especially when you come across something negative, you might feel depressed for days…*

[P4]

In the collectivist culture of Chinese families, illness was regarded as a shared family event. Family members would proactively intervene in the patient’s information-seeking behavior and act as an “emotional firewall” by discouraging the patient from searching for information. As a result, patients were protected and constrained by family emotions from the very beginning.

*My husband doesn't want me to search randomly online. He said, "Don't look at random stuff on the Internet. Just follow what the doctor says.*

[P6]

#### Problem-Solving–Oriented Information Search

The information-seeking behavior of participants revolved around a series of specific questions, reflecting their use of web-based resources as a vital supplement to inadequate clinical information. Through information retrieval, patients sought to enhance their sense of control over the disease, understand diagnostic outcomes and disease staging, explore treatment options, and support decision-making, while also addressing symptoms, side effects, and issues related to recovery and lifestyle adjustments. The participants described their experience of understanding disease staging through web-based information.

*I searched for it online. The doctor told me not to look it up, but I did anyway. T stands for the diameter, like the size of the tumor. So if it’s T2, that means it’s in category 2, right? And N1 means there’s lymph node metastasis. I couldn't get an answer from the doctor, so later, when I checked my discharge record, I saw that I'm T1N1M0, which means there’s no distant metastasis. After I found that out online, I was like, okay, I get it.*

[P17]

In the management of symptoms and side effects, information retrieval was also used to guide practical interventions.

*Mainly, I wanna search for the side effects of the injection. 'Cause I felt really awful after getting the red potion injected four times before. Every time, my stomach was killing me, and I'd throw up. So I wanna look it up online to see which time the side effects would be the worst and stuff. Then I'll see if the side effects will be less severe after I switch to a different medicine.*

[P6]

*Like, if my white blood cell count is low and I'm wondering what kind of medicine to take, or if I've got a bad taste in my mouth and my tongue is really white and thick and I wanna know how to relieve it, I'll just search for it online.*

[P3]

Several participants described that the brevity of clinical consultations often left their information needs insufficiently addressed, leading them to rely on web-based resources for additional medical explanations.

*You can see how busy the doctor is here. He can't talk to you for more than 10 minutes. He has so many patients, so there are many things you have to learn by yourself from other sources.*

[P7]

### Cognitive Judgments Amidst the Information Fog

#### Overview

When patients with breast cancer sought web-based health information across various platforms, they found themselves immersed in a landscape of multiple sources and diverse perspectives. This interweaving of information from different channels offered patients more clues for understanding their illness and treatment options, while also increasing the complexity of their cognitive judgment. Patients had to repeatedly weigh the authenticity of the information and its suitability for their specific disease stage and physical condition. When web-based information conflicted with existing knowledge or medical advice, they often experienced cognitive dissonance and indecision. Throughout this process, patients attempted to compare, filter, and reflect upon fragmented or conflicting information, assigning it personalized meaning in alignment with their own experiences.

#### Interweaving of Multichannel Information

Participants obtained health information through various web-based channels. Some initiated inquiries using general search engines, such as Baidu, and sought the truth by comparing multiple search results.

*Yeah, I used Baidu, no other search engines. I think it’s pretty reliable 'cause most of the stuff I saw was posted by doctors. Like which doctor from which hospital… For the same question, check out a few more results. If they're more or less the same, then it should be pretty accurate. Just search the same question a couple more times.*

[P6]

Some participants searched for real-life experiences shared by other patients on social media platforms, while remaining highly vigilant against advertisements and potential scams.

*Sometimes I also used Douyin. There are some people who are fellow patients, and they post videos directly on it. For example, like the Pyrrotinib I'm taking, they'll show its side - effects on Douyin, and it’s more vivid there… What’s on Douyin is a real reflection. Like some people do live - streaming to talk about their illness progress. They're quite honest and won't tell lies. Sometimes when I compare, it seems like I have the same symptoms too. Of course, some people might be just trying to sell stuff, so I stop watching after that…*

[P7]

Some participants also viewed artificial intelligence (AI) tools as efficient and objective sources for interpreting medical terms and integrating health knowledge.

*Besides the imaging data, I also had a carcinoembryonic antigen test. These are the two main factors, right? … I read on DeepSeek that it mainly looks at CA and CA153, 'cause these two things are characteristic of a high response to breast cancer. … My daughter checked both DeepSeek and ChatGPT for me, and the results were pretty much the same. … I think this stuff is great. It’s all about the data, and now AI can be trusted. Isn't there an AI hospital opened in Shanghai now?*

[P13]

Most participants tended to trust recommendations from doctors or hospital platforms, with their choices often determined by assessments of these channels’ authority, convenience, and personal usage habits.

*Can you still modify this interface? This was the essence of deep thinking. I don't often use Deepseek. I clicked it a few times, then stopped. Sometimes it got stuck, but once it got stuck, I couldn't get out of the interface.*

[P12]

*I think what Deepseek provides was a more objective perspective. They won't say something is definitely bad or good. There are no vested interests involved.*

[P11]

#### Judgment of Information Authenticity

In assessing the authenticity of web-based health information, participants often cast themselves as information detectives, proactively using various cognitive strategies to discern truth from falsehood. Confronted with complex sources and uneven-quality web-based content, patients attempted to mitigate potential risks through personal experience and repeated verification. Some participants noted that on platforms dominated by user-generated content, the authenticity of information relied heavily on individual judgment. Since these platforms featured both content based on genuine experiences and information driven by advertising or commercial promotion, participants had to continually discern between them while reading.

*On Xiaohongshu, you have to make your own judgment. Because there might be many that are relatively genuine, and there might also be some that are advertising-oriented, aiming to tell you what medicine to take or what to do. So they tend to write in that direction.*

[P11]

To enhance the reliability of their judgments, some participants cross-verified information by conducting cross-platform or multiple searches. This reflected their active efforts to strengthen confidence in the authenticity of information through consistency validation.

*For the same question, I would look at several results. If they are roughly the same, then they should not be too different. Search a few more times.*

[P6]

Additionally, some participants emphasized the authority of information sources and the scientific rigor of content, often choosing to remain cautious when a foundation of trust was lacking.

*What was said online can be quite confusing. You need to distinguish between what is true and what is false…Like those kinds of things on TikTok that are uncertain, where I don't have any real experience, or it’s not with friends or anything like that. I don't dare to try them; I don't dare to take any risks or give up the treatment here and try something else (on the internet), because the stakes are too high.*

[P2]

Several participants further developed verification strategies for authoritative personal accounts, reflecting patients’ critical stance toward superficial authority.

*First of all, I definitely need to check if this person is real… I'll look at his videos, the words he says to each patient. Where could he find so many shills? It’s impossible. So many different patients.*

[P14]

However, a portion of participants acknowledged that, when confronted with a complex information environment, their own judgment abilities were limited, which often left them feeling uncertain and powerless in verifying information.

*I'm not sure whether the information on the internet is good or bad. Some of it is correct, while some is incorrect.*

[P8]

#### Information Applicability Assessment

Upon obtaining information, participants typically did not adopt it immediately. Instead, they analyzed its applicability in light of their personal condition, treatment stage, and individual differences. One participant noted that when using generative AI tools, they did not directly follow the suggestions provided but rather treated them as supplementary reference material.

*DeepSeek really thought things through and gave more detailed advice. For example, when I asked about eating peaches, it warned me to be cautious. Then, based on that advice and my own situation—like whether I had mouth ulcers or diarrhea—I decided whether to eat them or not. So, if I had diarrhea that day, I knew not to eat peaches because they might make it worse. I appreciated how it reminded me of these things so I could choose whether it was okay to eat or better to avoid.*

[P18]

At the same time, participants recognized that web-based information was often based on group data and might not be fully applicable to individual circumstances. This awareness prompted them to maintain a certain distance from such information and to avoid applying it rigidly.

*Regarding the internet, it is also based on big data, right? It’s not an individual, either. For instance, how this person behaves might also be the case for you. It’s just a proportion or percentage of the big data…*

[P9]

Some participants prioritized seeking information that aligned closely with their personal needs, using this as the primary criterion for judging the information’s value.

*I won't browse random online information anymore. Whenever I come across any content suggesting a poor prognosis for triple-negative cases, I won't even bother to read it. I only look at those that are of the same type as mine and say the prognosis is relatively good.*

[P14]

Several participants mentioned that they adjusted the content of their information search based on their needs at different times in treatment.

*If after my radiotherapy is completed, how should I recover, how to exercise, learn to do sports, I will continue to search online, based on the actual situation. I need to change the search content according to my own disease development. For example, in the later stage, if I have just finished chemotherapy, I will start radiotherapy. What should I pay attention to?*

[P3]

Moreover, the assessment of information relevance demonstrated clear phase-specific characteristics. Several participants indicated that the content of their information-seeking was continually adjusted throughout the treatment process.

*After getting sick, I started searching for information about the disease online… I rested for half a year and didn't search again after returning to work. This time, the illness recurred, and I was more diligent in checking the information.*

[P1]

#### Cognitive Confusion in the Context of Information Conflict

When web-based health information conflicted with or contradicted treatment plans prescribed by their primary physicians, participants often experienced cognitive dissonance, which led to decision-making difficulties and a crisis of trust. In such situations, they had to repeatedly weigh conflicting advice from different authoritative sources, considerably heightening their psychological burden. Some participants described feeling confused and hesitant when they encountered discrepancies between web-based information and their doctors’ advice.

*The internet also says so. The doctors here say that radiotherapy is needed. Of course, there are doubts. Don't you think so? I'm just a patient, and I'm very confused. As long as someone says you don't have to have radiotherapy or chemotherapy, you'll be torn. It’s all like this, right?*

[P14]

*It’s still a very difficult decision-making process… The information on the internet is somewhat contrary to that of the doctor. It can affect the treatment plan your doctor gives you… Then your confidence in your attending doctor may decrease.*

[P4]

However, when web-based information conflicted with what their doctors said, patients often kept their doubts to themselves, for fear of challenging medical authority. This reflected patients’ reluctance to question professional authority within a high power-distance cultural context.

*I didn't tell the doctor I got the info online 'cause I'm afraid the doc’s gonna diss me. Like, the doc might say, "If you trust the internet so much, go ask those people online. Don't come to me.*

[P4]

The direct consequence of information contradiction was a widespread deadlock in choice and a psychological burden. This contradiction left patients feeling caught in the middle.

*Most doctors seem to say pretty much the same thing. But then it’s like, traditional Chinese medicine has its own way of looking at it, and Western medicine has another.*

[P2]

*It’s really tough to figure out what’s true and what’s not on your own—there’s just so much conflicting info out there.*

[P14]

Faced with decision-making difficulties, some participants would repeatedly verify and dwell on these contradictory pieces of information, reflecting their underlying desire for cognitive consistency.

*...I’ve looked online, and some of them talk about how it’s advanced and how it’s impossible to cure this disease anyway, that you live with cancer, but one of the points I’m struggling with right now is that some of them talk about how your body will definitely break down if you do chemo, and that’s 100% true. But if you don’t do chemo, and this disease just continues, which option is better?*

[P8]

#### Construction of Information Meaning

By integrating medical data with personal experiences, patients were able to derive new meaning from information. Dissatisfied with her doctor’s brief explanation, one participant independently conducted a detailed assessment, comparing earlier imaging reports and using AI tools to perform calculations.

*I myself have tested for carcinoembryonic antigen… Besides the imaging data, I also have the carcinoembryonic antigen. These are the two main factors, right?" … My daughter checked DeepSeek and ChatGPT for me, and the results were similar… We also calculated the volume… For example, for magnetic resonance imaging (MRI), I used MRI as a comparison… Then we calculated its volume… This indicates that the size has decreased… Overall, it has shrunk by 68.95%. I'm very happy that it’s reduced by two-thirds… This proves that my own thinking and reaction were correct.*

[P13]

After repeated comparisons and deliberation, participants developed a stable perception of the roles played by different information sources. One participant clearly distinguished the value of AI tools from that of doctors.

*Deepseek is just a reference sometimes. It might explain things in a more detailed and nitpicky way. Doctors, on the other hand, might just go through things quickly. If you don't understand, you can ask. Docs talk so fast that you might not get what they mean at all. You can ask Deepseek, and it'll give you a more detailed explanation. But like those indicators I asked you about last time, well, you know, professionals should do professional jobs. It can't replace doctors and nurses.*

[P18]

Some participants also constructed their own visions of the future based on the individual stories of their fellow patients.

*You know, when you're scrolling through stuff these days, there are a bunch of really well - known breast cancer doctors with their own personal accounts… If you come across the good cases they share, it'll have a positive impact on you. But sometimes, if you see the not - so - good stuff, it can mess with your mood. There’s this one doctor’s video. He said one of his patients was in a similar situation to me, and it seemed like the prognosis was okay. That patient also lived a pretty long time.*

[P4]

Through long-term engagement with information, patients gradually developed their own knowledge frameworks, transforming fragmented information into a coherent understanding of their illness.

*I’ve been looking up new drugs and treatments—’cause, let’s be real, everyone just wants to survive. When I first got diagnosed, I pretty much did all the research I could. I got a decent idea of the main treatment options—first-line, second-line meds, all that. So I kind of accepted, okay, I’ll go through those during this year of treatment. But then I started thinking—what happens after that? Will there be any new drugs by then? Any new treatment approaches I could try next?*

[P10]

Participants defined the process of information searching as an empowerment process, through which they enhanced their information literacy by means of continuous exploration and summarization.

*Searching for reading materials is also a form of learning and it helps us, y'know. I think it’s pretty good to have this kind of resource. At least it can guide me. Doctors are so busy taking care of so many patients. There’s no way they can cater to every single patient’s needs. Sometimes, you gotta rely on yourself.*

[P7]

*No matter how smart AI is, it’s not like it can read your mind. But I do think it’s something you have to learn how to use. The better you understand it, the more likely it is to give you the answers you actually want. I’m not super familiar with it yet—like, I haven’t really studied it in a structured way or anything. But now there are more and more tools out there, like DeepSeek and other platforms, to help people learn.*

[P11]

Through ongoing exploration, comparison, and summarization of information, these participants gradually built a firm understanding of their disease, treatment options, and personal responses. They subsequently translated this knowledge into actionable guidance for both decision-making and daily life.

### Adaptation Under the Pressure of Web-Based Information

#### Overview

Facing the vast and complex web-based health information, patients with breast cancer have developed various adaptation strategies to alleviate the cognitive load, emotional stress, and decision-making difficulties brought about by the information. These strategies mainly fall into 4 categories: translating information into actions, emotional regulation and coping, establishing support networks, and acceptance and expectation adjustment.

#### Transform Information Into Action

This subtheme illustrated how participants translated acquired knowledge into concrete daily actions after obtaining, filtering, and verifying web-based health information. This process strengthened their self-management capacities and improved communication with health care providers. Some participants mentioned modifying their diets based on recommendations from tools like DeepSeek, tailoring the information to their individual health conditions.

*Like, Deepseek says you gotta be careful when eating peaches. If you've got oral ulcers or diarrhea, don't eat 'em. So I'll judge based on how I'm feeling today. If I'm having diarrhea, I know not to eat 'em. But if my appetite’s normal, no oral ulcers, and I don't feel nauseous, then I can eat 'em. So I can choose whether to eat 'em or not according to its suggestions.*

[P18]

In information-driven self-care practices, some participants proactively integrated traditional Chinese health preservation wisdom, regarded as a crucial action resource, into their daily health management.

*I watched a video on Video Account about the Three-Bean Drink. It seems pretty good. I mean, it’s basically a food therapy, you know?*

[P18]

In medical communication, participants drew on web-based information to engage with their physicians in seeking personalized guidance. This practice demonstrated how patients translated external information into concrete actions and interaction strategies, thereby obtaining a sense of psychological reassurance and decision support through such exchanges.

*I saw someone on Xiaohongshu saying that taking Sanguisorba Tablets can increase white blood cell count. So I went to ask my doctor if I could take those pills. I was like, "Doc, I saw this on Xiaohongshu, and I even took a screenshot." Then I showed it to my doctor.*

[P12]

Participants further integrated web-based rehabilitation information into their daily training routines, achieving a phased transition from cognition to action. Through practice, they continuously tested and adjusted the applicability of the information while cultivating a sense of self-efficacy. This process reinforced the connection between information empowerment and tangible rehabilitation outcomes.

*Sometimes people on the Internet tell me to do exercises. My hands have recovered better than most people because I do exercises well. They tell me what exercises to do online, and I do them for 40 minutes. I jog for 20 minutes and do exercises for another 20 minutes. Actually, I stick to it every day. On the third and fourth days, I start with 10 minutes, then 15 minutes, and then 20 minutes. I gradually increase the time.*

[P3]

#### Emotional Regulatory Coping

Faced with vast and often complex web-based health information, participants used multiple strategies to regulate their emotions, alleviate anxiety, and restore psychological equilibrium. These strategies encompassed both the active pursuit of positive information and the conscious limitation of exposure to content in order to mitigate the negative emotions elicited by web-based sources. For instance, some participants sought psychological comfort by deliberately reviewing positive content and reading about the recovery experiences of fellow patients.

*I think the cure rate for breast cancer was relatively low before. I saw online that the survival period is now over five years, and the cure rate has reached several tens of percent. I think the current cure rate is quite high. I think this boosts my confidence… Because I have triple-negative breast cancer. But I saw that many of my friends with triple-negative cancer, as long as they survive for these five years, basically, they won't have a recurrence. This gives me a lot of confidence.*

[P3]

Additionally, some participants maintained emotional stability through self-motivation and psychological self-regulation. They not only regulated their emotions by acquiring information but also reinforced positive feelings and beliefs through intentional behavioral practices.

*I will browse the internet… I will copy some good sentences to encourage myself. Now, even a doctor shouting slogans has become quite famous on the video platform. On it, I have also learned to shout slogans. This mental victory method can change my inner emotional state.*

[P13]

A small number of participants also halted their information retrieval, reduced their search frequency, and avoided specific platforms or negative topics to mitigate the emotional interference caused by web-based information.

*I've searched before, and I don't do it anymore now. Now… even if I search, it won't be of any use.*

[P5]

*But the more I searched, the more anxious I became, and the more I didn't want to look.*

[P1]

For some of the participants, the cell phone was never far away, and they could only reduce searching or reading web-based information by diverting their attention. However, there were also participants who had to passively accept web-based information.

*I don't even want to search anymore. I just watch some funny videos every day.*

[P 2]

*Anyway, it’s just for some gaming… Sometimes, it would come up on TikTok by itself, but I still wanted to check if there were any similar things related to me, like what others use for treatment. Just to see what others are posting on TikTok… Without searching, it just shows up on TikTok.*

[P1]

#### Build a Support Network

Participants sought assistance from others within complex information environments to alleviate their decision-making pressure and emotional burdens.

*I joined those patient groups. Then I'd listen to what they said and also do some searches on the group. There’s a lot of info there. You know, I just wanna learn more about it.*

[P17]

Patients often referred to the experiences of fellow patients and sought decision-making advice from doctors or nursing staff, or had family members screen and verify information on their behalf.

*Seeing this information, one would surely be confused about how to choose this path and which one to take… But my daughter will always help me make the right decision.*

[P8]

*I talked to the doctor about online info. Like, I asked if I could have radiotherapy for bone metastases. He said I didn't need it and should just listen to doctors. Doctors have seen more cases. What I've seen is just one-sided.*

[P7]

#### Acceptance and Adjustment of Expectations

Through information acquisition and practical experience, some participants gradually adjusted their expectations regarding treatment outcomes, shifting their focus from defeating the disease toward managing specific symptoms and maintaining daily functioning.

*I just checked online, and some info said it’s in the advanced stage. Anyway, it’s impossible to cure your illness. You gotta live with this cancer…*

[P8]

Upon recognizing the gap between their own knowledge and the professional system, participants learned to delegate decision-making authority to professional authorities after thoroughly exploring information.

*Anyway, when you're in a tough spot trying to make a decision, I always think doctors have more experience than you, right? You've been looking up info for just a few months, but doctors have seen tons of patients. You gotta trust them as much as you can.*

[P4]

## Discussion

### Summary of Results

The findings of this study authentically reflect the web-based information-seeking experiences of patients with breast cancer, which can be summarized under 3 core themes: drivers of information seeking, cognitive judgments amid information fog, and adaptive mechanisms under web-based information pressure. These themes are not isolated categories but rather depict a dynamic psychosocial process: how patients navigate a challenging digital information environment from their initial needs to ultimately develop personalized coping strategies.

### Findings

This study found that information-seeking behavior among patients with breast cancer is driven by both emotional and problem-solving needs. The anxiety and uncertainty brought by a diagnosis drive emotional information-seeking centered on seeking reassurance, while simultaneously prompting problem-solving searches to understand the disease and participate in decision-making. Consistent with the classification of web-based help-seeking behaviors of patients with cancer in previous studies, the study by Feng et al [29] mainly focused on web-based health communities and categorized patients’ help-seeking behaviors into informational and emotional support needs. This study further expands this perspective by extending the scope of analysis from help-seeking behaviors in web-based community interactions to a broader context of OHIS. Notably, these 2 drivers do not switch off and on simply; they often coexist and reinforce each other. This finding resonates with research by Liu et al [30], based on a dual-factor model, which confirmed that pediatric patients’ OHISs are simultaneously influenced by complex factors—both facilitators (such as perceived stress) and inhibitors (such as learned helplessness), revealing the inherent tension within behavioral drivers. In the Chinese context, this driving force is further moderated by the family system. Phenomena observed in this study, such as spousal discouragement and proxy searching by children, align with Feng’s [31] findings that Chinese family communication patterns influence information-seeking behaviors undertaken for family members or oneself. This highlights how, within collectivist cultures, disease management is often viewed as a shared family unit event.

This study reveals patients’ cognitive judgment processes within uncertain information environments. Patients develop strategies such as cross-verifying multiple channels, tracing information sources, and assessing applicability to construct personal cognitive safety nets amid information fog. The core paradox of this process lies in the dual role of information: while it may enhance patients’ sense of control over their illness, excessive web-based information, poor quality content, and conflicting viewpoints can also trigger decision-making difficulties, diminished trust in information, and persistent anxiety [32,33]. This study particularly highlights the critical role of doctor-patient communication within the information fog. When patients conceal their web-based doubts for fear of challenging medical authority, conflicting information becomes internalized as a psychological burden. This finding strongly corroborates the study by Mahmood et al [34], which demonstrated that patient-centered communication significantly reduces frustration during information-seeking among cancer survivors. It cannot be overlooked that while OHIS has to some extent challenged the traditional paternalistic medical model, its role in clinical practice is increasingly being reinterpreted. Far from threatening physician authority, it may actually enhance the depth and quality of doctor-patient communication [35]. Furthermore, patients’ heightened focus on dietary adjustments and nutritional therapies when evaluating information is deeply rooted in local health beliefs and traditional practices. This provides empirical support for moving beyond universal health literacy models and constructing culturally sensitive analytical frameworks.

In contrast to the results of the review by Chen et al [8], our study found that Chinese patients with breast cancer did not use the websites of national or nonprofit cancer organizations to search for health information. Chen et al [8] showed that patients with breast cancer, including those in China, often relied on these websites for information. This disparity may indicate that the patients in this sample have relatively low digital health literacy and have limited access to information through professional web-based channels. Digital health literacy specifically refers to a series of technical abilities and cognitive skills that individuals need to discover, understand, evaluate, and apply web-based health information [36]. The patients with breast cancer in this study’s reliance on social media and general search engines may directly indicate their deficiencies in the 2 key aspects of digital literacy, namely identifying the authority of information and conducting effective web-based navigation. In this context, digital health care approaches, such as hospital official apps, web-based patient communities, or intelligent question-answering systems, can provide patients with alternative channels for information acquisition, thereby reducing the obstacles caused by insufficient digital literacy [37,38]. The demand of patients with cancer for digital health functions is closely related to their digital health literacy levels, and there are also differences in the preferences for digital health interventions among patients with different literacy levels. This indicates that digital literacy is an important factor influencing the use of digital nursing tools [39]. Through these platforms, patients can not only access timely and reliable health information but also pose questions, verify information, and receive personalized advice in an interactive environment, which in practice contributes to enhancing their ability to understand and apply information. Meanwhile, research indicates that there are significant disparities in digital health literacy among breast cancer patients. Patients with low literacy levels face greater difficulties in participating in eHealth interventions, highlighting the need to consider patients’ literacy levels when designing digital nursing resources [40].

One of our findings is that the web-based information search behavior of patients with breast cancer changes with the disease stage, similar to the results of Madge, but their research focused on patients who had undergone breast cancer surgery [41]. Our participants had a wider range of disease stages, including both patients who had just undergone breast cancer surgery and those with recurrent breast cancer. The phased changes in information search behavior were more significant, suggesting that web-based health information intervention should be time-sensitive.

What is worth noting is that many participants with breast cancer, when searching for web-based health information, not only used traditional search engines and social media but also showed a high preference and trust in AI platforms. As shown in related studies, while traditional web-based resources remain dominant, LLM-based chatbots are becoming a resource for health information for some users, especially those who are younger and have a higher level of trust in AI [42]. However, this AI preference also comes with potential risks. The study found that the web-based health advice provided by AI models contains information misguidance and inaccurate information sources [43]. It can be seen that AI preference may trigger new cognitive trust traps. Therefore, we suggest that when promoting AI-assisted information services, it must be incorporated into the collaborative doctor-patient communication process, and through educational courses, web-based lectures, etc, improve patients’ digital health literacy, thereby enhancing the accuracy of information acquisition and decision-making quality for patients [44].

### Theoretical Extensions in Web-Based Health Information Contexts

This study’s findings align with key processes in the stress-coping theory, thereby providing empirical support for understanding this theory in digital health information contexts. Concurrently, this study theorizes the complex web-based health information environment itself as a significant digital stressor, thereby expanding the application context of stress and coping theories. Findings indicate that for patients, this environment serves not only as a repository of resources for seeking support but may also constitute a persistent source of psychological stress due to information overload, contradictions, and uncertainties. In essence, an overwhelming or inconsistent volume of information can trigger anxiety and stress responses, potentially leading to avoidance behaviors [45]. Research findings also indicate that information avoidance and reduced retrieval frequency are not merely passive reactions, but rather contextualized emotion regulation strategies. This discovery complements traditional coping classifications’ understanding of avoidance strategies. Unlike the original theory’s focus on immediate coping, this study found that patients construct meaning around their illness through repeated comparison and integration of information from multiple sources, thereby adjusting their expectations for the future. This demonstrates the long-term and developmental nature of the coping process. It is worth noting that since this theory was not originally designed for digital health information scenarios, some new phenomena related to web-based information conflict and digital uncertainty lack direct conceptual counterparts within the theory. The findings of this study provide new perspectives on the applicability and extension of this theory within digital health information environments.

### Limitations

This study has the following limitations. Although most participants came from different cities and prefectures within Hunan Province of China, there are differences in language, economic development, and urban-rural structure within the province. However, the sample size of the qualitative research is limited, and it may still not cover the diversity of digital literacy and socioeconomic backgrounds. Participants were patients from a specialized cancer hospital, and these findings may not fully reflect the experiences or perspectives of health care providers in other medical systems or regions with differing technological infrastructure. Although semistructured in-depth interviews were adopted to obtain data, the respondents’ recollections might have a selective bias, and some emotional or behavioral experiences might not be accurately expressed or recalled. At the same time, qualitative research itself has a strong interpretative and subjective nature, and different analysts might form different understandings of the same material. This study was conducted during a treatment stage of patients with breast cancer and did not longitudinally track the web-based information search process and behaviors of patients with breast cancer. Future research could further focus on the differences in information coping patterns among people from different cultures and with different health literacy levels, and also examine how coping strategies evolve dynamically over the course of the treatment from a longitudinal perspective. This study analyzed the research results based on the theory of stress and coping. It provided theoretical support for understanding web-based information-seeking behavior. However, these theories may have limitations in terms of applicability in the specific cultural context of China. Future research could further validate these theories by integrating cultural adaptation theories.

### Implications of Research Findings for Policy and Future Research

This study reveals the complex experiences of patients with breast cancer in navigating web-based health information in the digital age. These findings not only deepen our understanding of patients’ health information behaviors but also provide significant insights for improving health policies, optimizing clinical practices, and guiding future research directions. The key points are as follows:

1. Promote the construction of a health information support system: Health policy-makers and medical institutions should take the lead in building national or regional official disease information and support platforms. These platforms should integrate evidence-based medical guidelines, treatment pathway descriptions, side-effect management, and rehabilitation knowledge and present them in easily understandable forms such as videos and interactive question-and-answer sessions to reduce information risks.
2. Incorporate patient information behavior management into routine nursing and doctor-patient communication norms: It is recommended that modules for assessing and coaching patients’ health information literacy be added to nursing practice guidelines and doctor-patient communication training. Health care providers should proactively inquire about patients’ information sources and concerns and provide tailored information prescriptions. They should also guide patients in translating web-based information into safe, actionable self-management behaviors.
3. Conduct digital health literacy education: In the future, health information literacy education courses should be developed for patients with cancer. Special attention should be paid to digitally vulnerable groups such as older people and those with low educational levels, and barrier-free digital health literacy intervention measures should be designed to promote fairness in health information access.
4. Deepen local research and interdisciplinary cooperation: Future research should explore in depth how culture-specific factors shape information behavior patterns and carry out interdisciplinary cooperation among nursing, psychology, information science, and computer science. In the future, intelligent health information question and answer and decision-making assistance tools based on AI and in line with the Chinese cultural context can be developed.

### Conclusions

The web-based information-seeking experience of Chinese patients with breast cancer is a dynamic process involving motivation, cognition, and adaptation. Their search behavior stems from dual needs: emotional drive and problem-solving. Within a complex and conflicting information environment, they assess authenticity and applicability while constructing meaning. Through strategies such as information transformation, emotional regulation, support network building, and expectation adjustment, they achieve individual adaptation. Theoretically, this finding enriches the research framework of digital health literacy and information behavior and provides empirical evidence for understanding patients’ coping mechanisms in a complex information environment. In practice, this study suggests that clinical and nursing staff should pay attention to patients’ emotional needs, cognitive judgment abilities, and adaptation strategies when designing digital health interventions and information support strategies. They should provide multichannel, easy-to-understand, and actionable information resources and combine social support and emotional regulation guidance to help patients obtain, understand, and apply health information more effectively.

## Supplementary material

10.2196/80363 Checklist 1 COREQ checklist.

## Abbreviations

AI: artificial intelligence
COREQ: Consolidated Criteria for Reporting Qualitative Research Checklist
OHIS: online health information search
